# Supplementary material for: The Compound (E)-2-Cyano-N,3-diphenylacrylamide (JMPR-01): A Potential Drug for Treatment of Inflammatory Diseases
Source: Pharmaceutics. 2022 Jan 13;14(1):188. doi: 10.3390/pharmaceutics14010188 (PMC8777680; doi:10.3390/pharmaceutics14010188)
Supplement: Supplementary file 1 [file pharmaceutics-14-00188-s001.zip › pharmaceutics-1535598-supplementary.pdf]

pharmaceutics

Supplementary Materials: The Compound (*E*)-2-cyano-*N*,3-diphenylacrylamide (JMPR-01): A Potential Drug for Treatment of Inflammatory Diseases

Pablo Rayff da Silva, Renan Fernandes do Espírito Santo, Camila de Oliveira Melo, Fábio Emanuel Pachú Cavalcante, Thássia Borges Costa, Yasmim Vilarim Barbosa, Yvnni M. S. de Medeiros e Silva, Natália Ferreira de Sousa, Cristiane Flora Villarreal, Ricardo Olímpio de Moura and Vanda Lucia dos Santos

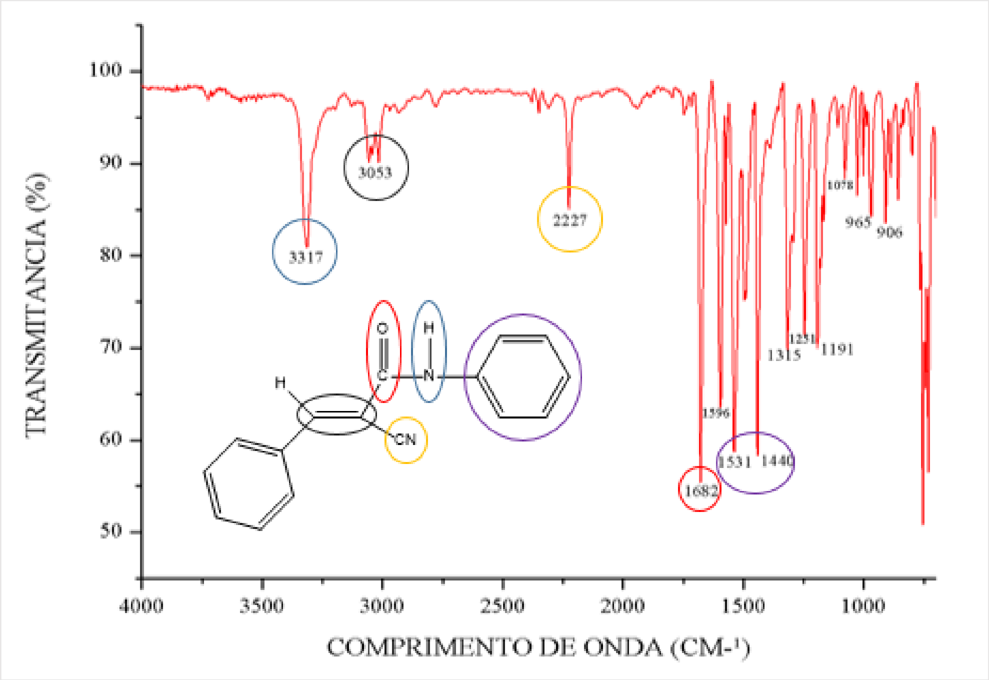

Figure S1. FT-IR spectrum of JMPR-01.

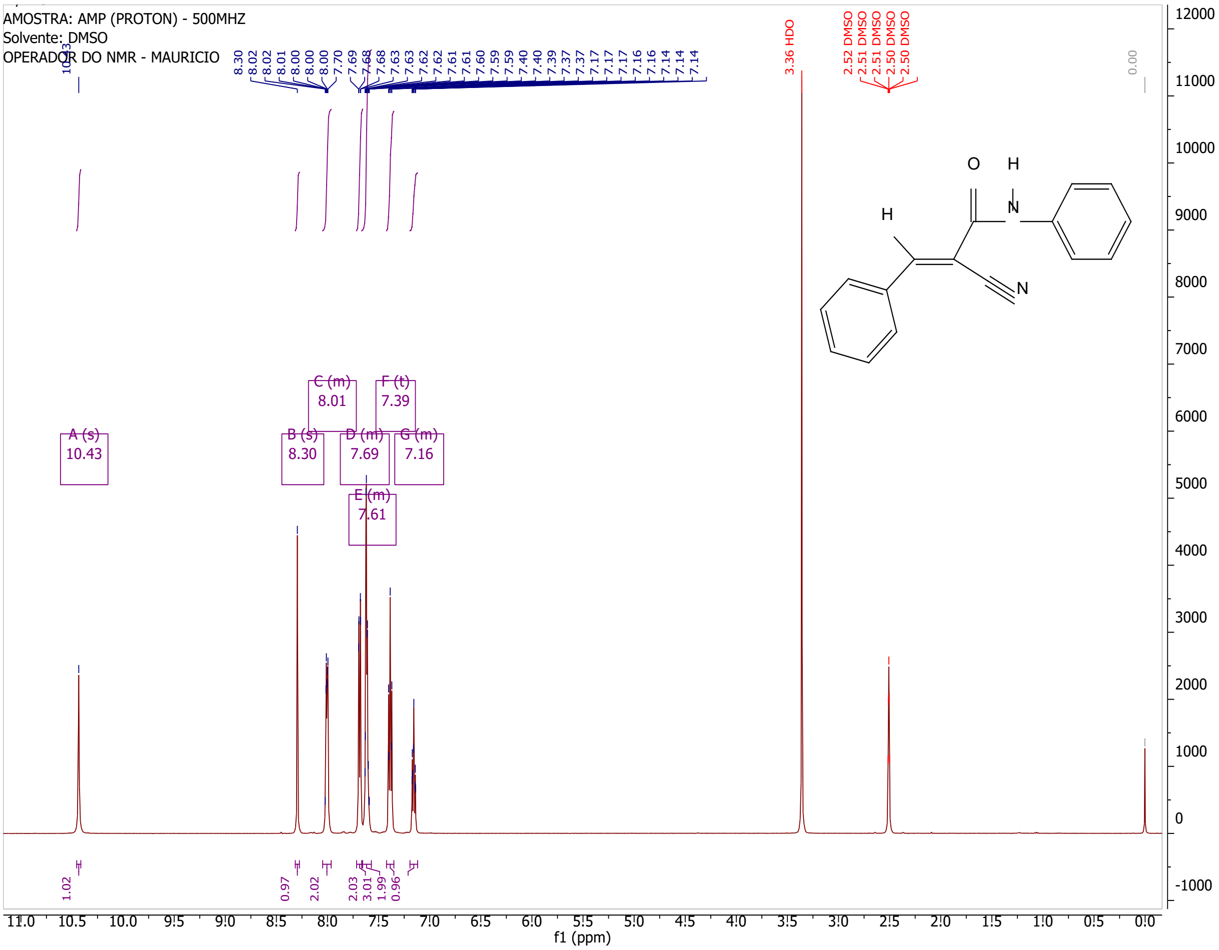

Figure S2. ^{1}H NMR spectrum of JMPR-01.

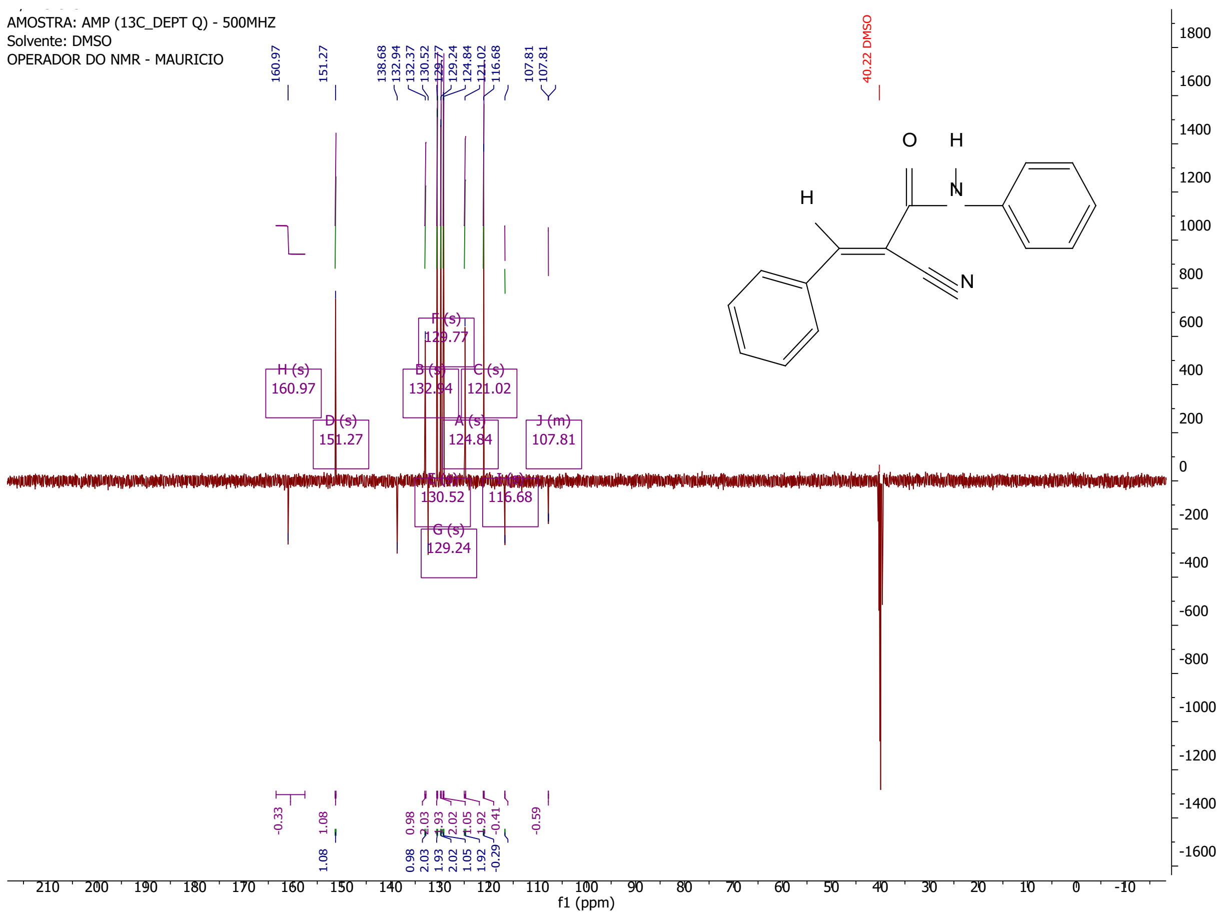

Figure S3. ^{13}C NMR spectrum of JMPR-01.

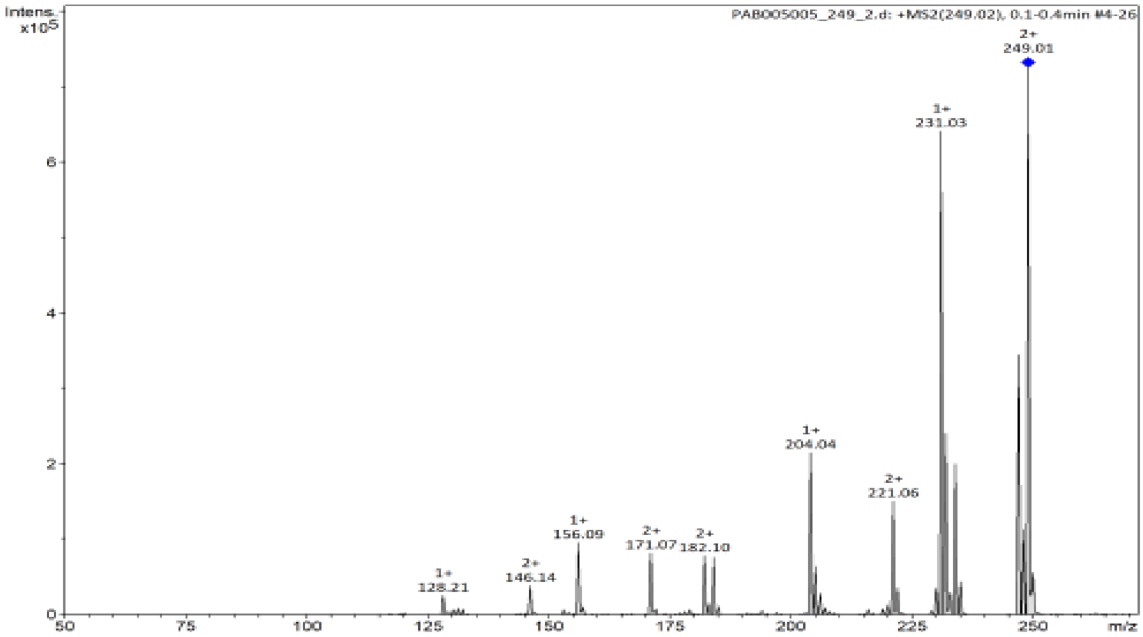

Figure S4. HRMS *m*/*z* [M[+] + H] of **JMPR-01.**

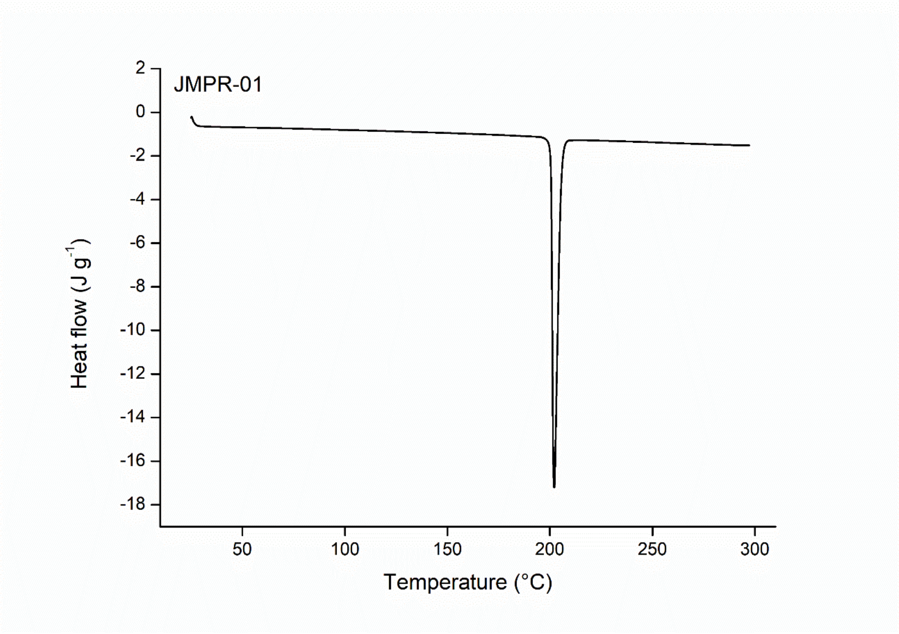

(A)

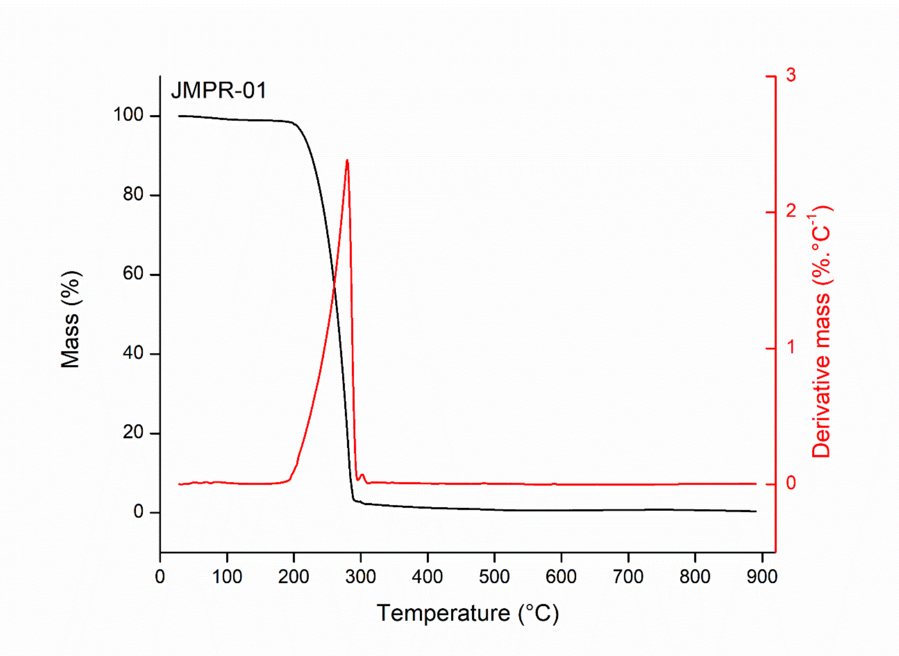

(B)

Figure S5. Differential Scanning Calorimetry (DSC) (**A**) and Thermogravimetric Analysis (**B**) of **JMPR-01**.

Table S1. CC_{50}, EC_{50} and Selectivity Index of JMPR-01 standardized by TNF-α inhibition in determined in cultures of J774 macrophages.

JMPR-01	Mean (± S.D.)	Selectivity Index (S.I.)
**CC_{50}** (μM)	977.25 (± 655.36)	139.20
**EC_{50}** (μM)	7.02 (± 4.24)	
